# Dental diaspora: oral health care attitudes and experiences in culturally and linguistically diverse mothers in Australia

**DOI:** 10.1186/s12913-022-08708-6

**Published:** 2022-11-03

**Authors:** Kanchan Marcus, Madhan Balasubramanian, Stephanie D Short, Woosung Sohn

**Affiliations:** 1 Population Oral Health, Faculty of Medicine and Health, The University of Sydney Dental School, Surry Hills, NSW Australia; 2 Menzies Centre for Health Policy and Economics, School of Public Health, The University of Sydney, Camperdown, NSW Australia; 3 Health Care Management, College of Business, Government and Law, Flinders University, Bedford Park, SA Australia

**Keywords:** Oral health, Cultural Diversity, Diaspora, Health workforce, Health Services accessibility

## Abstract

**Background:**

Globally oral health care is unequally accessible or utilised within culturally and linguistically diverse (CALD) migrant communities. Yet much remains unknown about CALD mothers and their oral healthcare experiences in Australia. Hence, this paper explores the oral health care attitudes and experiences of CALD mothers within the Australian context with the broader objective to reduce oral health inequalities.

**Methods:**

Qualitative semi-structured interviews were conducted from a social constructivism paradigm. Participants were foreign country born, spoke language/s other than English and have a child. Purposive snowball sampling and recruitment was conducted through CALD organisations and social media. Participants were interviewed for their attitudes and experiences to dental care and frequency of utilisation in Australia and the home country. Interviews were transcribed verbatim and grounded analysis (Strauss and Corbin) performed. Researcher bias was reduced through reflexivity and triangulation.

**Results:**

The participants (*n* = 33) included 20 CALD mothers born in India and 13 from either China, Fiji, Nepal, Macedonia and Israel. The theme, *experiences with health workforce personnel* revealed positive attitudes toward CALD providers from similar cultural and/or linguistic backgrounds. We coin these CALD providers as the ‘dental diaspora’. The dental diaspora facilitated CALD mothers through culture and/or language factors, alleviating cost barriers and flexibility in appointments. Dental travel to the home country was affirmed, however family visitation was the foremost reason for travel.

**Conclusion:**

The findings suggest that the dental diaspora plays a significant role in promoting oral health care utilisation for first generation CALD mothers in Australia. This paper brings to light the phenomenon of the ‘dental diaspora’ as an essential health workforce that contributes to addressing inequities in oral healthcare utilisation within CALD migrant communities. Universal health coverage in oral health is further affirmed, as aligned to the WHO policy context.

## Background

Widening health care inequities are evident across and within countries, resulting in complex sociocultural, economic, political and other challenges [1]. Comparably, these inequities apply to oral health care, whereby a delay in dental care results in poor health outcomes which is further associated with non-communicable diseases [2, 3]. Pragmatically, oral health prevention requires a shift from individual responsibility and instead commands interconnected upstream policies that consider social, historical, cultural, environmental and other dynamics [4, 5]. Sustainable Development Goals accentuate (1) good health and well-being and (2) reducing inequalities to be achieved by 2030. Subsequently, the WHO resolution on oral health care affirms in reducing oral health care disparities, with an emphasis on Universal Health Coverage (UHC), prevention and multisector primary care [6].

The evidence illustrates oral health inequalities and inequities in the mouth for women and culturally and linguistically diverse (CALD) mothers [7]. Nettleton analysed intersections between Western governments, dental providers and dental agents - the mother, which affirmed power-knowledge relations [8]. In this, the mouth is an indicator of inequality with the *type of* mother acting as agents upon their children’s dental health practices [8]. However negative discourses ‘blaming’ a mother for children’s poor oral health is ill conducive. Despite evidence that mothers are influential in children’s oral health care behaviours [9], the level of agency a mother has, is compounded by multiple inter-related barriers in provider experiences, social factors, time, cost and pregnancy among others [10, 11]. This is further compounded for CALD mothers, who encounter language, literacy, racial and further barriers [7]. For instance, an integrated mixed method systematic review on the oral healthcare barriers and facilitators was undertaken in CALD carers. This review identified complex upstream, mid-level and populations level factors, with numerous studies conducted in the USA and only one in Australia [7]. Thus, context specific solutions are required to reduce population inequalities. For example Arora et al. conducted a pre-post oral health literacy study. They interviewed Vietnamese mother-infant dyads from disadvantaged geographic areas [12]. Findings affirmed a need for oral health care information to be provided in native languages. However, understanding further enablers beyond information translation, within country-contexts is needed, given the multicultural diversity of CALD populations and lack of UHC globally [13].

Population outcomes with dental health care providers from CALD backgrounds is poorly understood. In 2012, Marino and colleagues identified the need for transcultural skills and cultural competence of dental students in terms of providing care to diverse populations in Australia [5]. This study underlined systemic failures in attracting diverse ethnicities into dental education and factors impacting CALD population access to oral health, with the potential for cultural matching of health professionals [5]. In the USA, a national survey of Hispanic and Latino dental workforce was conducted [14]. Findings affirmed that 85% of Hispanic/Latino dentists spoke Spanish within their clinical practice and 40% specifically supported underserved populations [14]. Further integrated models of oral and general health is emphasised within dental education and the health profession, which requires context specific solutions to population needs [15]. Yet historic disparities and under representation of minority students in dental education hinders opportunities to advance equity in CALD populations [16]. These studies highlight the significant need for a diverse oral health care workforce and integrated models of care to reduce barriers in oral health care access and population inequities [17].

A small body of literature recognises health care inequalities/inequities in CALD mothers and populations [7, 18, 19]. For example, a scoping review identified CALD migrant mothers were less likely to access healthcare, with barriers identified in ‘appropriateness’ of services [20]. The literature evidences the need for professional interpreters from CALD backgrounds or increased diversity within the workforce, reflecting cultural and/or religious norms with patients [5, 21]. These studies however are not specific to oral health in CALD mothers within the Australian context and evidence with provider experiences is largely absent. In Australia, there are one in four practising migrant dentists [22] and evidence of dental underutilisation in CALD adults [23]. The Australian Bureau of Statistics reports over seven million migrants in 2020, more than 300 languages identified [24, 25] and a lack of UHC in oral health, albeit cost barriers, which also prevents accessibility [6, 13]. What remains unknown is whether oral health care providers are meeting the specific needs of CALD mothers. In this manner, qualitative evidence promotes an opportunity to extend ‘why’ and ‘how’ health care improvements can be realised [26]. Therefore, using a qualitative approach, this study aims to explore the oral health care attitudes and experiences of CALD mothers within the Australian context. Importantly, diversity in voices and the lived experiences of CALD mothers is needed to inform the evidence base to support and implement co-designed solutions to improve population equity.

## Method

The epistemological basis of this study is drawn from a social constructivism paradigm in line with Vygotsky, which encompasses historical, social and cultural contexts of individuals [27]. Knowledge is contextualised and socially constructed with multiple forms of reality [27]. Interpretive modes were utilised, through open ended questions with participants, which enabled free responses. The researcher interpreted participant responses based on the contextual meanings described. Further, the researcher adopted a shared space between the etic-emic approach (also referred as insider-outsider) [28]. From this perspective, the emic stance was illustrated with mutual language and culture with ten participants. This is advantageous in allowing the researcher to understand cultural nuances and develop trust. Yet, divergent language and culture from an etic perspective occurred for majority of participant interviews. To reduce bias for etic-emic interviews, self-reflexivity was undertaken throughout the research which included journaling, reflection writing and mind maps to identify the researchers own preconceptions and beliefs [29]. Engaging in transparent findings with the broader research team also occurred during data collection and analysis, which added reliability. Relatedly, the principal investigator, who conducted all interviews, is a CALD migrant Australian female researcher, with considerable qualitative research experience. Bilingual translators were not utilised, although this option was made available to all participants. Pseudonyms were randomly selected from online cultural/ethnic websites. Quotes utilised in this paper were chosen to reflect the nature of majority of responses and participant diversity.

### Participant recruitment and sampling

Studies confirm that foreign born, non-English speaking adults underutilise dental services [23] however understanding why this is the case is insufficient in the literature. To explore this gap, we focus on mothers as an influential group for childhood oral health behaviours [30]. In the Australian context, research, policy and government employ ‘CALD’ terminology to describe ethnic and migrant populations [31]. Noting limitations to this terminology, CALD mothers were included if they were foreign country born, from a non-English speaking country and conversed in a language other than English. Our ‘CALD’ classification is also sustained by an epidemiological study by Pham and colleagues [32]. Participants had at least one child under 12 years at the time of recruitment. Additionally, this migrant CALD population is considered first generation immigrants, in the Australian context. A purposive snowball sampling strategy was utilised, whereby participants could disseminate study details to others. Participants were initially recruited through cultural and religious community outreach organisations using contact details from websites. However, this method proved unsuccessful. A second strategy wherein social media flyers were disseminated to the administrators of cultural Facebook pages and community language schools. Administrators had the discretion of passing study details onto members. Ninety percent of the sample were recruited this way and 10% were recruited through snowball sampling via word of mouth.

Study recruitment and interviews were conducted during April-July 2021. Interview questions were largely open ended, with semi-structured questions to steer the discussion. Questions focused on the attitudes and experiences to dental care and frequency of utilisation in Australia and the home country. For instance, probing questions included how to find or access dental services, utilisation experiences, barriers and facilitators to accessing oral healthcare, traditional remedies and any dental problems experienced. This method allowed for individualised flexibility in participant experiences. Participant information and consent was distributed, highlighting voluntary participation. Telephone and/or zoom was utilised for interviews, and only one interview was conducted face-face as per participant convenience. Interviews were conducted either late evenings or over weekends. Duration of interviews ranged between 20 and 55 min depending on participant discussions. All participants once lived in the Greater Western Sydney region of New South Wales, although two were currently residing in Victoria and Canberra. Greater Western Sydney is multiculturally diverse, whereby 42.9% of the population are overseas born [33]. Participants migrated to Australia either for marriage, study or via the family stream. Interviews were audio recorded using an Olympus digital recorder and transcribed verbatim to Word.

### Data analysis

Thematic analysis using grounded approaches based within the school of Strauss and Corbin was conducted [26]. A constant comparative approach was used to code and explain the data. Initial coding encompassed the barriers, facilitators and experiences, that is, open coding [26]. A second stage of theoretical coding was undertaken to investigate the relationships between concepts. Consequently, this led to sorting multiple codes (such as provider support, ethical practices) into categories, which was initially grouped as ‘workforce’ and ‘barriers to care’. Memos and diagrams were written to track analytical connections and meaning between coded data. Themes were then conceptualised using an iterative process and critical team feedback, which was then represented in a conceptual diagram. Theoretical sensitivity allowed data to be explained through memos, analytical insights during the interviews, and the process of coding [26]. Thus, understanding what was happening in the data. Further, researcher bias was reduced through open ended questions, repeated examination of the data, self-reflexivity using journaling, mind maps and memos [26].

 Ethical approval was granted by The University of Sydney Human Research Ethics Committee (2021/101). Participation was voluntary and informed consent was provided prior to interviews. Confidentiality was maintained by non-identifying personal details and participants had the right to withdraw from the study at any time, without reason. Consent was provided by all participants and a $20 store voucher was gifted to all participants for their valuable time (this was also presented to participants that withdrew).

## Results

Four CALD mothers were excluded as they either opted out prior to the interview or were Australian born. Data saturation was reached by thirty interviews, whereby no new relationships or findings were revealed [34]. The resultant eligible sample (*n* = 33) included CALD mothers born in India (20) and (13) were from China, Fiji, Nepal, Macedonia and Israel (see Table 1). Analysis resulted in two overarching themes: dental hesitancy and experiences with health workforce personnel. For the purposes of this paper, we explicate the second theme.

Our study revealed that five CALD mothers, who resided in Australia for over 10 years, did not utilise any regular dental care. A further five CALD mothers, residing in Australia for less than 10 years, had a lack of dental visitation. Key reasons included cost, lack of time or need and navigating the healthcare system. This suggests that access or utilisation of oral healthcare services is impacted by various factors, beyond acculturation (that is, the number of years in the host country as a form of cultural integration into host country norms), for the CALD mothers in this study.

**Table 1:** Demographics of participants

Country of birth	China	*n* = 4
	Fiji	*n* = 5
	India	*n* = 20
	Israel	*n* = 1
	Macedonia	*n* = 1
	Nepal	*n* = 2
Languages spoken	Cantonese/Mandarin	*n* = 4
	Fiji-Hindi	*n* = 5
	Gujrati	*n* = 2
	Hebrew	*n* = 1
	Hindi	*n* = 10
	Macedonian	*n* = 1
	Marathi	*n* = 2
	Nepalese	*n* = 2
	Punjabi	*n* = 5
	Tamil	*n* = 1
	Telegu	*n* = 2
Years migrated to Australia	< 10 years > 11 years	*n* = 14 *n* = 19
Gender of dental provider matters	Doesn’t matter Female preferred	*n* = 30 *n* = 3
Visited CALD provider in Australia	Yes No	*n* = 17 *n* = 16
Dental travel to home country at least once	Yes No	*n* = 14 *n* = 19

Participant confidentiality maintained

### Experiences with health workforce personnel

#### Dental diasora

Based on the findings of this study, we build upon the work of previous scholars Marino et al. on cultural matching by coining the term ‘dental diaspora’. We define the term ‘diaspora’ using a multidisciplinary integrated analysis, as reported by Grossman. Hence, the *diaspora refers to a transnational community or group, who dispersed or emigrated from the original homeland, but remain socially identified with that identity* [35]. In this manner, providers were either from the same or similar birth country, shared linguistic characteristics or from similar world regions for example, South Asia.

Participants affirmed positive attitudes toward the dental diaspora. CALD mothers reported cultural comfort and/or communication that fostered trust and rapport. Two mothers substantiated the importance of conversing in native languages with the dental provider to assist with medical terminology translation. Intergenerational language differences were highlighted in families for grandparents who live in the household with mothers and children. An example of this intergenerational difference between a CALD mother and her Australian born, ethnically diverse child is highlighted in the quote below. The importance of multilingual staff was helpful, to converse in English with the Child and Mandarin with the parent or grandparent.

…*And um, she got a um, the um, assistant, that speaks English and she can also well explain it to my son because they (son) speak English, they don’t really understand Mandarin. So I think that’s the good combination. Yeah, cause my, m-my son, they will speak, speak English. They don’t quite understand that, it’s other way around. (Xiang, China born).*

The dental diaspora alleviated specific oral health care utilisation issues which included waived fees, extended opening hours and flexibility in appointments for CALD mothers. CALD mothers reported that the gender of the oral health care provider *didn’t matter at all* except for three mothers. Most dental care experiences were described as ‘respectful’. Reiterated several times were ‘waived gap fees’ by CALD providers, which meant the mother *was not going to incur any cost as well*. This was appreciated by CALD mothers, a few who reported seeking the same provider for this reason. CALD mothers reported that CALD providers extended clinic after hours, to accommodate appointments for CALD mothers, including opening a clinic on Sunday for the whole family. This generally occurred with CALD providers who had seen the mother/family previously.

*I have gone to a lady (CALD dentist) who’s done after five appointments for me, because I was in pain. So, she opened up longer for me. (Shreya, Fiji born).*

Three mothers reported negative experiences with the dental diaspora from a similar cultural and/or linguistic background in Australia. For these participants, the concern was either related to the quality of treatment or unethical practices. In one example ‘the fillings fell out within the year’. This issue was further perceived that dental providers ensured return patients for business/profit purposes. Upon explanation, one CALD dentist suggested multiple return appointments for three fillings. In this instance, the dentists’ request didn’t make any logical or practical sense to the mother. In circumstances of negative experiences or unmet dental care needs, two solutions were highlighted by CALD mothers. Firstly, the preference was an insurance provider, which was considered trustworthy as promoting patient health care. Noted here was that the insurance dental provider diaspora did not matter. The second approach to accessing oral health care services was that mothers awaited (in pain or managed the pain) for dental care treatment to be received in the home country by trusted providers. Eight mothers affirmed that dental care in Australia is less focused on patient health care outcomes, and rather on business profits.

*She kept, you know, calling me again and again on these different appointments. Telling me the stories that if I take this wisdom tooth out, this will happen, that will happen and she charged me. She didn’t do anything. I was suffering with pain…Now, you come for the, uh, X-rays, Okay. Now, she, you come for an, uh, explanation. But she took five appointments, charge me above $200, $150 every appointment…(Jasleen, India born).*

Several participants received dental care in the home country (prior to COVID), which was essentially from providers of shared cultural and linguistic background. Reasons for this varied, however participants confirmed that the travel was notably for family visitation, and dental treatment was a secondary consequence. Dental costs were also key factors, which was more affordable in the home country. Travel to the home country further promoted family support, with carers for children, familiarity of the health care system and/or cultural comfort of known providers, which facilitated dental care for mothers. COVID has caused interruption to oral health care services for a quarter of CALD participants who would have otherwise travelled to the home country for family and consequently received oral health care.

*…I have been to Nepal uh, 3 years back now, almost 3 and a half years back, so uh, that time I had uh, my scaling done and a thorough check-up for my oral uh, health. So my friend did it and she said everything is fine. No, according to the dentist’s point of view, she said everything is fine… (Rita, Nepal born).*

*It was a good, quite good experience (India)…Yeah she was - was from my locality. From my community. So it was very convenient for me to visit her. (Sujathaa, India born).*

#### Primary health care providers

Primary health care workers specifically mentioned as general practitioners and nurses and midwives facilitated oral health care for CALD mothers and their children. This was described as the first point of contact in the healthcare system for general oral health information and/or health care communication …*uh, my, I have to ask my GP because, uh, she’s yet to provide me, uh, she will refer me to a nearby dentist (Keerthy, India born)*. In one example, a community nurse informed the mother (once she had a baby) about the dental care needs of the child using the Blue Book (that is, a personal record from birth to 4 years to check the health and development of children born in NSW; available in 18 languages), *the nurse mentioned to me during mother’s group (Li na, China born)*. Suggestions were made to improve the dental information in this Blue Book by using graphics and simple pictorial information on how to brush or care for children’s teeth. Yet sometimes providers of primary health care missed an opportunity to promote oral health care for CALD families. (Possibly due to ever increasing workloads of nurses and doctors). For instance, a handful of participants suggested that the GP should have informed the mother of oral health care issues upon migration. This is because the delay in dental care worsened problems in the mouth over time.

*…Again it flows to you from your general practitioner. So, if they advise you…on those um, about those facilities, yes that is very helpful because they are our first point of contact when we go and visit probably in couple of months time and um, apart from it, we usually don’t go and see a doctor until unless there is an issue (Vaishali, India born).*

**Fig. 1:**
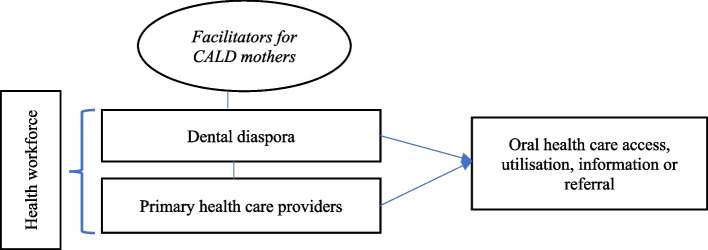
Conceptual findings of CALD mother’s experiences with health workforce personnel for oral health care

Figure 1 conceptually draws upon the findings from of our study. Moreover, the dental diaspora promotes oral health care for CALD mothers. In conjunction, primary health care providers support CALD communities as the first point of contact in the healthcare system. Transdisciplinary collaboration with dental and primary health care providers is required to integrate and promote oral health equitably across population groups.

## Discussion

Findings from this qualitative study underlined the significant role of the dental diaspora supporting CALD mothers in Australia. The support was expressed through positive attitudes and experiences in language and/or culture, waived costs and flexibility in appointments. Importantly too, primary healthcare providers were key enablers for oral health care promotion, which either referred mothers to dentists or distributed information, including the Blue Book. Further, this study is aligned to the WHO resolution on oral health which endorses collaboration between the dental and primary health workforce to improve population health care equity. Caution however is required with interpretation. Variability between CALD mothers, in terms of education, literacy, socioeconomic and sub-cultural differences were evident. Furthermore, mothers chose to conduct the interview in English, and thus displayed a level of English proficiency. Whereas new migrant mothers, without English language proficiency could reveal differing needs, one of which may include a stronger preference for providers who communicate in the same language as the CALD mother.

Positive attitudes and experiences were disclosed by CALD mothers with the dental diaspora. This was demonstrated in first generation CALD migrant mothers through the cultural and/or linguistic comfort, alleviation of fees and appointment flexibility. Consequently, beneficial health impacts from the dental diaspora is aligned to the evidence. For example, Mertz and colleagues confirmed that Hispanic/Latino dentists were more than twice as likely to treat diabetic patients, support patients with low literacy, accept public insurance and converse in non-English languages [14]. Further, respectful, affirmative dental encounters validate repeat visitation. In addition, a survey analysis reported that migrant dentists affirmed positive work experiences in Australia [22]. In contrast to our qualitative findings, a cross-sectional analysis with diverse dental students was conducted by Marino and colleagues. This multivariate study found similarities between ethnic and Australian dental students on the basis of value orientation theory (human nature, people nature, time, activity and relationships) [36]. Interestingly cultural language was upheld by majority of the ethnic dental students and while University students differ in acculturation levels, our qualitative findings offer a distinct perspective, in which CALD mothers had positive experiences with the dental diaspora. Notably, policymakers cannot rely on the dental diaspora alone, to negate multiple, complex oral health care inequities experienced by CALD communities. In addition, accountability and regulation challenges are affirmed in the literature, with post dental complications associated with dental tourism [37]. This occurrence was reported due to cost, cultural factors and family support, which may continue to drive dental care to be received in other countries. This reinforces the need for UHC in oral health, as well as integrated care to support CALD communities and their oral health care in Australia [38].

Previous studies demonstrate the siloed nature of the oral health care profession which is transitioning towards integrated primary health care in Australia [15]. Accordingly, the first point of contact for migrants within the health care system is primary health care providers. Namely, CALD mothers reported the general practitioner and community nurses and midwives, which is thus an opportune time to advance oral health care for migrant CALD communities. A ‘Watch me grow study’ in Sydney, conducted by Garg et al. substantiated that CALD parents attend general practitioners for all their children’s health care needs. Preference for doctors from the same cultural or linguistic background was further verified [39]. Furthermore, a qualitative study identified the need for collaborative care across the health sector to address social determinants [15]. Thus, our findings align with the evidence, encouraging further integration of oral and general primary health care [17].

Notably, the contributions from this study offer dental experiences and insights within the multicultural Australian health care context, which is largely lacking in the literature. Key strengths of the paper include data saturation, self-reflexivity [29] and firsthand recount of oral health care experiences in this population. Although, with a diverse group of CALD mothers, this study does not adequately reflect the views and experiences of all CALD mothers. Transferability is also not appropriate with qualitative studies in specific context - samples, however the study provides important insights in dental access and utilisation for CALD communities. Our sampling method also highlights the inequitable opportunity to participate in this study, whereby flyers were distributed online or through community channels, therefore CALD mothers without membership to these groups were inadvertently excluded from the sampling and recruitment process. Slightly over half the group comprised mothers with tertiary qualifications, indicating a high level of knowledge or access to resources, information or employment networks. We acknowledge that the dental diaspora is skewed with specific ethnic communities who attain high levels of education and thus greater numbers of dental students from diverse backgrounds should be supported to reflect the multicultural Australian population. Findings could differ in groups with varied levels of English proficiency, particularly refugee groups. During recruitment, multiple efforts were made to differing ethnic groups, including Samoan, Arabic and Vietnamese networks, however this approach was unsuccessful, respectively. A community health worker further assisted with the distribution of study details to CALD parenting groups, however COVID-19 lockdowns occurred, which halted this process. Further qualitative research should consider acculturation [19], socioeconomic and immigration impacts in oral health. Importantly, findings represent CALD voices thereby contributing to the body of knowledge in oral health equity, migrant populations and provider experiences.

## Conclusion

The present study examined oral health care appropriateness and relevance for CALD mothers. Our study highlights positive attitudes and experiences with the dental diaspora, as reported by first generation CALD migrant mothers. This included cultural and/or linguistic factors, waived costs and appointment flexibility. Inequalities in access or utilisation of dental care is further championed through key health sector gatekeepers, specifically the GP and community nurses. The dental diaspora is an essential health workforce that could stem inequalities and inequities in oral and general health for CALD communities. Subsequently, this study supports primary health sector integration, as aligned to UHC and the WHO resolution on oral health care.

## Data Availability

The datasets used and/or analysed during the current study are available from the corresponding author on reasonable request however, all the data generated or analysed during this study are included in this published article.

## Abbreviations

CALD: culturally and linguistically diverse
NSW: New South Wales
UHC: Universal Health Coverage
WHO: World Health Organisation
